# The ratio of skeletal muscle mass to visceral fat area is a main determinant linking circulating irisin to metabolic phenotype

**DOI:** 10.1186/s12933-015-0319-8

**Published:** 2016-01-20

**Authors:** You-Cheol Hwang, Won Seon Jeon, Cheol-Young Park, Byung-Soo Youn

**Affiliations:** Department of Medicine, Division of Endocrinology and Metabolism, Kyung Hee University Hospital at Gangdong, Kyung Hee University School of Medicine, Seoul, Republic of Korea; Department of Internal Medicine, Division of Endocrinology and Metabolism, Kangbuk Samsung Hospital, Sungkyunkwan University School of Medicine, Pyung-Dong, Jongro-Gu, Seoul, 110-746 Republic of Korea; Biomedical Research Center, Ulsan University Hospital, University of Ulsan College of Medicine, 877 Bangeojinsunwhando-ro, Dong-gu, Ulsan, 44033 Republic of Korea

**Keywords:** Irisin, Skeletal muscle, Visceral fat, Glucose tolerance, Insulin sensitivity

## Abstract

**Background:**

The aims of this study were to investigate whether circulating irisin is associated with favorable metabolic parameters and how the association differs according to body composition in humans.

**Methods:**

A total of 424 subjects (233 men and 191 women), aged 23–73 years (mean age 47.1 years), were enrolled from the Seoul Metro City Diabetes Prevention Program. Body composition was determined using an impedance body composition analyzer, and serum irisin level was measured using a commercial kit.

**Results:**

Serum irisin was correlated with favorable metabolic parameters including less obese, lower blood pressure and glucose levels and healthy lipid parameters. The skeletal muscle mass to visceral fat area ratio (SVR) was positively correlated with the serum irisin concentration (*r* = 0.10, *P* = 0.04). When the study subjects were divided into tertiles according to their SVR, serum irisin was correlated with favorable metabolic phenotypes in those subjects in the upper tertile. However, there were no such correlations in the lower tertile. In addition, serum irisin was inversely related to pre-diabetes/type 2 diabetes (T2D) independent of other risk factor for T2D and insulin resistance [OR (95 % CI); 0.66 (0.49–0.90), *P* = 0.009].

**Conclusions:**

The compositions of skeletal muscle and visceral fat play key roles in the association between circulating irisin and a patient’s metabolic phenotype.

**Electronic supplementary material:**

The online version of this article (doi:10.1186/s12933-015-0319-8) contains supplementary material, which is available to authorized users.

## Background

Adipose tissue is an active endocrine organ. Recent studies have suggested that liver and bone regulate whole body energy homeostasis through their production of hepatokines [1] and osteocalcin [2], respectively. Skeletal muscle also communicates with other organs through its secretion of hormones called myokines, which are mostly released into the blood stream during or immediately after physical activity [3–6].

Irisin is a novel myokine that is, proteolytically processed from the product of the FNDC5 gene. Both exercise and the peroxisome proliferator-activated receptor gamma coactivator 1α (PGC1α) induce irisin expression. Irisin induces the browning of subcutaneous adipocytes and thermogenesis by increasing the level of uncoupling protein 1 (UCP1) level. In doing so, irisin mediates the beneficial effects of exercise on energy metabolism. In one animal study, 3 weeks of wheel running increased plasma irisin levels in mice, and adenoviral FNDC5 overexpression in high-fat diet-induced obese mice showed increased oxygen consumption, body weight loss, improved glucose tolerance, and lower fasting insulin compared to those of diet-induced obese control mice [3].

However, the associations between circulating irisin and physical activity or glucose metabolism are still elusive in humans. Initial reports demonstrated a two-fold increase in circulating irisin levels after 10 weeks of endurance training in healthy adults [3]. However, a subsequent study found that muscle FNDC5 mRNA expression was only increased in elderly subjects after endurance training, and not in younger subjects [7]. In a cohort of 118 patients, the FNDC5 mRNA expression in muscle was not correlated with glucose homeostasis [7]. Another study, however, found that serum irisin level was lower in patients with type 2 diabetes (T2D) than it was in subjects with normal glucose tolerance [8, 9]. Serum irisin was also found to be negatively correlated with fat mass, fasting glucose and dyslipidemia but not with other adipokines. Furthermore, irisin decreased during an oral glucose tolerance test (OGTT) in subjects with all degrees of glucose tolerance [10]. In contrast, another report showed that baseline irisin level was positively correlated with body mass index (BMI), blood pressure, fasting glucose, triglycerides, and insulin resistance [11].

Therefore, the aims of this study were to investigate whether circulating irisin is independently associated with favorable metabolic parameters including glucose tolerance and to determine whether the association between circulating irisin and metabolic profile is different from body composition which was determined by skeletal muscle mass to visceral fat area ratio (SVR), one of the two sources of circulating irisin.

## Methods

### Study population

A cross-sectional study was performed involving 424 subjects (233 men and 191 women) aged 23–73 years (mean age 47.1 years). The subjects were enrolled in the second year of the Seoul Metro City Diabetes Prevention Program (SMC-DPP) between August and December 2010. The SMC-DPP is a community-based follow-up program that is composed of three groups, including healthy people and subjects with pre-diabetes or T2D. The participants were recruited from six community health centers in Seoul. After excluding participants with previously diagnosed diabetes or anti-diabetic medications, a 75 g-OGTT was conducted in the remaining participants. The participants were classified into groups based on the result of the OGTT, in accordance with the diagnostic criteria from the American Diabetes Association. Subjects with a history of type 1 diabetes, secondary diabetes, or other problems such as major cardiovascular disease, hemodialysis, and malignancy were excluded. This study was approved by the Institutional Review Board at Kangbuk Samsung Hospital. Informed written consent was obtained from each subject.

### Anthropometric and laboratory assessments

Blood pressure was measured in duplicate after at least 5 min of rest, and the results were averaged. BMI was expressed as body weight in kilograms divided by the square of height in meters (kg/m^2^). Body composition was determined using the multi-frequency impedance body composition analyzer with eight-point tactile electrodes (InBody 720; Biospace, Seoul, Korea). The analyzer measured resistance at specific frequencies (1, 5, 50, 250, 500 kHz, and 1 MHz) and reactance at specific frequencies (5, 50, and 250 kHz). The following other measurements were also made: skeletal muscle mass (kg), body fat mass (kg), percent body fat (%), waist-hip ratio and visceral fat area (%). Additional data used in the analysis included arm circumference, arm muscle circumference, and basal metabolic rate.

Blood samples were collected after overnight fasting. Plasma glucose was determined by the hexokinase method using a Beckman glucose analyzer II (Beckman Instruments, Fullerton, CA, USA). High-performance liquid chromatography was used to measure HbA1c (Variant II, Bio-Rad Laboratories, Hercules, CA, USA). Total cholesterol, triglyceride, HDL cholesterol, and LDL cholesterol levels were determined by an enzymatic colorimetric assay using an autoanalyzer (Siemens, Tarrytown, NY, USA). High-sensitivity C-reactive protein levels were measured by immune-nephelometric assay using a BNII nephelometer (Dade Behring, Deerfield, IL, USA). Serum insulin level was measured using an immunoradiometric assay (Biosource, Belgium).

The homeostasis model assessment of insulin resistance (HOMA-IR) was calculated as the product of fasting serum insulin (mU/L) and FPG (mmol/L) concentrations, divided by 22.5. HOMA-B % was calculated using (20 × fasting serum insulin)/(FPG −3.5), and used to represent ß-cell function [12]. In this study, T2D was defined by the presence of one of the following: (1) FPG ≥7.0 mmol/L; (2) treatment with oral hypoglycemic agents or insulin therapy; or (3) 2 h post-load glucose ≥11.1 mmol/L. Normal glucose tolerance was defined as FPG <5.6 mmol/L and 2 h post-load glucose <7.8 mmol/L. Pre-diabetes is comprised of two different categories of impaired fasting glucose and impaired glucose tolerance. Impaired fasting glucose was defined as FPG of 5.6–6.9 mmol/L, while impaired glucose tolerance was defined as 2-hour post-load glucose of 7.8–11.0 mmol/L [13]. Metabolic syndrome was defined using the criteria proposed by the National Cholesterol Education Program Adult Treatment Panel III [14].

The serum irisin level was measured using an enzyme linked-immunosorbent assay (ELISA) commercial kit (AdipoGen, Korea). The intra-assay coefficients of variation (CVs) for irisin ranged between 4.86 and 8.19 %, and inter-assay CVs ranged from 8.03 to 9.72 %.

### Statistical methods

All data are presented as means ± SDs or proportions, except for skewed variables, which are presented as medians (interquartile range, 25–75 %). One-way ANOVA, followed by Tukey’s *posthoc* test, and the Kruskal–Wallis test were used to compare the differences between the irisin tertiles. A linear-by-linear association χ^2^-test was used to perform trend analysis between the tertiles. The spearman correlation coefficients were calculated to compare the irisin and clinical and laboratory measurements. Multiple logistic regression analysis was used to calculate an odds ratio (OR) for the presence of pre-diabetes/T2D. The results of the analyses are presented as OR with a 95 % confidence interval (CI). All statistical analyses were performed using PASW version 18.0 (SPSS, Chicago, IL, USA). *P* values <0.05 were considered statistically significant.

## Results

Baseline characteristics of the study subjects according to serum irisin tertile are shown in Table 1. The mean age decreased from the lower to upper serum irisin tertiles and there was no difference by sex. Waist circumference was inversely correlated with serum irisin level (*P* = 0.04). In addition, HOMA-IR decreased as serum irisin level increased. Serum irisin concentrations were significantly higher in subjects with normal glucose tolerance compared to subjects with pre-diabetes or T2D (2.16 ± 0.59 μg/mL in normal glucose tolerance and 1.77 ± 0.91 μg/mL in pre-diabetes or T2D, *P* < 0.001; data not shown). The irisin levels were also significantly higher in subjects without metabolic syndrome compared to those with metabolic syndrome (1.91 ± 0.92 μg/mL without metabolic syndrome and 1.78 ± 0.80 μg/mL with metabolic syndrome, *P* = 0.03; data not shown).

**Table 1:** Baseline characteristics according to serum irisin tertile

	Total (n = 424)	1st tertile (n = 142)	2nd tertile (n = 141)	3rd tertile (n = 141)	*P*
Irisin (μg/mL)	1.71 (1.44, 2.02)	1.35 (1.20, 1.45)	1.72 (1.62, 1.81)	2.30 (2.04, 2.68)	<0.001
Age (year)	47.1 (9.2)	48.7 (8.5)^a^	47.5 (8.7)^ab^	45.2 (10.0)^b^	0.005
Male (%)	233 (55.0)	88 (62.0)	71 (50.4)	74 (52.3)	0.11
Body mass index (kg/m^2^)	24.7 (3.5)	25.1 (2.9)	24.6 (3.9)	24.3 (3.6)	0.15
Waist circumference (cm)	84.3 (9.7)	85.8 (7.7)^a^	84.2 (10.7)^ab^	82.9 (10.4)^b^	0.041
Systolic blood pressure (mmHg)	121.3 (14.1)	123.1 (14.1)	120.6 (14.1)	120.1 (14.0)	0.18
Diastolic blood pressure (mmHg)	75.8 (10.2)	77.0 (10.8)	76.4 (9.7)	74.1 (9.8)	0.05
OGTT (mmol/L)					
0 min	6.4 (1.9)	6.7 (2.0)	6.3 (1.5)	6.3 (2.0)	0.13
120 min	8.6 (3.8)	8.8 (4.1)^ab^	8.0 (2.7)^a^	9.3 (4.4)^b^	0.041
A1c (%)	6.0 (1.3)	6.0 (1.1)	5.9 (1.0)	6.2 (1.6)	0.06
HOMA-IR	2.26 (1.61, 3.24)	2.50 (1.83, 3.26)^a^	2.30 (1.79, 3.23)^a^	1.90 (1.25, 3.40)	0.001
HOMA-B (%)	73.2 (52.4, 97.2)	74.2 (52.3, 99.1)	74.4 (54.7, 98.0)	72.2 (46.9, 92.9)	0.47
Type 2 diabetes (%)	92 (21.7)	30 (21.1)	29 (20.6)	34 (24.1)	0.54
Pre-diabetes/type 2 diabetes (%)	333 (785)	129 (90.8)	118 (83.7)	86 (61.0)	<0.001
Total cholesterol (mmol/L)	4.96 (0.90)	5.11 (0.85)^a^	5.00 (0.86)^ab^	4.77 (0.96)^b^	0.005
Triglyceride (mmol/L)	1.74 (1.25, 2.59)	1.45 (1.01, 2.26)^a^	1.25 (0.98, 1.70)^a^	1.08 (0.76, 1.44)	<0.001
HDL cholesterol (mmol/L)	1.31 (0.32)	1.27 (0.27)	1.34 (0.37)	1.34 (0.29)	0.09
LDL cholesterol (mmol/L)	3.03 (0.85)	3.11 (0.81)	3.05 (0.81)	2.93 (0.93)	0.22
High-sensitivity CRP (mg/L)	0.04 (0.02, 0.11)	0.04 (0.02, 0.09)	0.04 (0.02, 0.11)	0.05 (0.03, 0.11)	0.07
Metabolic syndrome (%)	155/414 (37.4)	59/137 (43.1)	54/138 (39.1)	42/139 (30.2)	0.027

Data are expressed as means ± SDs, medians ± inter-quartile ranges or frequency (%)

^a,b^The same letters indicate no statistical significance

With regard to body composition, there was a strong correlation between visceral fat area and skeletal muscle mass (*r* = 0.25, *P* < 0.001; data not shown). Skeletal muscle mass adjusted for visceral fat area was positively correlated with serum irisin concentration (*r* = 0.10, *P* = 0.042). However, there were no correlations for the total fat mass or percentage of body fat (Table 2).

**Table 2:** Correlation of irisin with Inbody measurements

	*r*	*P*
Waist circumference	−0.11	0.019
Waist to hip ratio	−0.15	0.002
Skeletal muscle mass	−0.06	0.20
Total fat mass	−0.02	0.69
Body fat %	0.02	0.67
Visceral fat area	−0.13	0.009
Skeletal muscle mass/visceral fat area	0.10	0.042
Arm circumference	−0.10	0.048
Arm muscle circumference	−0.09	0.08
Basal metabolic rate	−0.06	0.22

The study subjects were then divided into tertiles according to the SVR. In the upper tertile, Serum irisin was inversely correlated with age, obesity, diastolic blood pressure, glucose intolerance, HOMA-IR, and dysmetabolic lipid profile. However, there were no such favorable correlations with serum irisin in subjects in the lower tertile. In contrast, in these subjects, there was a positive correlation between the serum irisin and glycated hemoglobin levels (Table 3). In addition, we used waist circumference as an estimate for visceral abdominal fat instead of visceral fat area by InBody and divided the subjects into tertiles according to skeletal muscle mass to waist circumference ratio. Similar with the result form to skeletal muscle mass to visceral fat area tertiles, serum irisin level was inversely correlated with unhealthy metabolic profiles, especially in subjects in the upper tertile. However, there were no favorable correlations with serum irisin in subjects in the lower tertile (Additional file 1: Table S1).

**Table 3:** Correlation with serum irisin according to skeletal muscle mass to visceral fat area tertile

	Total (n = 424)	Lower tertile (n = 142)	Middle tertile (n = 141)	Upper tertile (n = 141)
Age	−0.17^**^	−0.06	−0.18^*^	−0.17^*^
Body mass index	−0.10^*^	0.05	−0.06	−0.22^**^
Waist circumference	−0.11^*^	0.01	−0.10	−0.20^*^
Systolic blood pressure	−0.11^*^	0.10	−0.20^*^	−0.14
Diastolic blood pressure	−0.14^**^	0.10	−0.22^**^	−0.25^**^
OGTT (0 min)	−0.25^***^	0.13	−0.21^*^	−0.52^***^
OGTT (120 min)	−0.00	0.11	−0.09	−0.07
A1c	−0.05	0.17^*^	−0.06	−0.27^**^
HOMA-IR	−0.18^***^	0.14	−0.20^*^	−0.37^***^
HOMA-B %	0.01	0.02	0.04	0.04
Total cholesterol	−0.16^**^	0.02	−0.14	−0.27^**^
Triglycerides	−0.21^***^	0.09	−0.25^**^	−0.37^***^
HDL cholesterol	0.13^**^	−0.02	0.13	0.21^*^
LDL cholesterol	−0.10	−0.03	−0.09	−0.20^*^
High-sensitivity CRP	0.12^*^	0.16	0.11	0.14

^*^ *P* < 0.05, ^**^ *P* < 0.01, ^***^ *P* < 0.001

Finally, we determined whether serum irisin level was independently associated with the presence of abnormal glucose tolerance. In this analysis, log transformed serum irisin was inversely associated with pre-diabetes/T2D independent of age, sex, BMI, waist circumference, systolic blood pressure, triglycerides, and HDL cholesterol [OR (95 % CI); 0.60 (0.45–0.79), *P* < 0.001; model IV]. Further adjustment of HOMA-B % [OR (95 % CI); 0.55 (0.41–0.75), *P* < 0.001; model V] or HOMA-IR [OR (95 % CI); 0.66 (0.49–0.90), *P* < 0.01; model VI] did not affect this association between serum irisin and pre-diabetes/T2D (Table 4).

**Table 4:** Multiple logistic regression analysis of log transformed serum irisin for pre-diabetes or T2D

	OR (95 % CI)	*P*
Model I	0.49 (0.37–0.64)	<0.001
Model II	0.55 (0.42–0.72)	<0.001
Model III	0.56 (0.42–0.74)	<0.001
Model IV	0.60 (0.45–0.79)	<0.001
Model V	0.55 (0.41–0.75)	<0.001
Model VI	0.66 (0.49–0.90)	0.009

Model I: no adjustment

Model II: Model I + adjustment for age and sex

Model III: Model II + adjustment for body mass index, waist circumference, systolic blood pressure

Model IV: Model III + adjustment for triglyceride, HDL cholesterol

Model V: Model IV + adjustment for HOMA-B %

Model VI: Model IV + adjustment for HOMA-IR

## Discussion

In the current study, higher circulating irisin was correlated with favorable metabolic profiles, including waist circumference, blood pressure, blood glucose level, lipid parameters, and HOMA-IR, a marker of insulin resistance. With regard to glucose tolerance, circulating irisin was inversely correlated with the presence of pre-diabetes/T2D, independent of other well-known risk factors for T2D, including age, sex, BMI, waist circumference, systolic blood pressure, and atherogenic lipid profiles. In particular, serum irisin was still protective against abnormal glucose tolerance after further adjusting for HOMA-B % or HOMA-IR.

To date, the biological function of irisin has been largely unknown, with contradictory results in human studies [3, 7–11, 15, 16]. In agreement with our results, serum irisin level was found to be significantly lower in patients with new-onset T2D compared with subjects with normal glucose tolerance, and increased irisin level was associated with reduced risk for T2D independent of BMI, renal function, diastolic blood pressure, HOMA-IR, and triglyceride levels [8]. In addition, a previous study found that circulating irisin was negatively correlated with BMI, percent fat mass, and waist to hip ratio and positively correlated with insulin sensitivity. Moreover, circulating irisin concentration was decreased significantly in subjects with T2D [15]. In contrast, another report showed results in striking contrast to our results [11]. In that report, baseline irisin level was significantly higher in subjects with metabolic syndrome compared to subjects without metabolic syndrome and was positively correlated with unhealthy metabolic phenotype. In addition, irisin was independently associated with HOMA-IR and ten-year risk of cardiovascular disease after adjustment for confounders. Therefore, it is possible that irisin secretion is increased in adipose/muscle tissue and/or there is a compensatory hyper-secretion of irisin in order to overcome irisin resistance in obese subjects.

Low SVR may represent a state of sarcopenic obesity, which has been reported to be associated with insulin resistance and metabolic syndrome [17–19]. In addition, a previous study demonstrated that lower SVR was independently associated with the presence of metabolic syndrome and arterial stiffness [19]. In our study, circulating irisin was not correlated with skeletal muscle mass (*r* = −0.06, *P* = 0.20); however, visceral fat area was inversely correlated with circulating irisin level (*r* = −0.13, *P* < 0.01). Subjects were then divided study into tertiles according to SVR. Although circulating irisin was inversely correlated with BMI, waist circumference, unhealthy metabolic profiles, and insulin resistance in the total subjects, the correlations were quite different according to SVR tertile. That is, although irisin level was strongly and inversely correlated with the aforementioned parameters in subjects in the upper tertile, as SVR decreased, the inverse correlations weakened. In the lower tertile, there were no favorable effects of irisin on metabolic profile. In contrast, there was no difference in serum irisin levels between the lower tertile and upper tertile (data not shown). We further analyzed the correlation between serum irisin and metabolic parameters according to the presence of metabolic syndrome (Additional file 1: Table S2). As a result, serum irisin was inversely correlated with BMI, waist circumference, fasting glucose, HbA1c, total cholesterol, triglyceride, LDL cholesterol, and HOMA-IR in subjects without metabolic syndrome. However, these correlations did not exist in subjects with metabolic syndrome. Therefore, it is possible that the function of irisin is more important than is the circulating irisin level per se with regard to metabolic effects, and body composition of skeletal muscle and visceral fat areas play key roles in its functionality. That is, circulating irisin appears to be dysfunctionally altered in subjects with lower skeletal muscle mass and higher visceral fat.

This study has several limitations. First, it was based on a cross-sectional analysis; thus, it is not possible to determine causal relationships. In particular, FNDC5/irisin is known to be released into circulation mainly during or immediately after physical activity, and it would be valuable to determine the association between incremental circulating irisin levels after exercise and its metabolic effects. Second, we did not measure FNDC5 gene expression from muscle or adipose tissue. We also did not measure the levels of other circulating myokines or adipokines, which may co-secrete from muscle or adipose tissue. Third, the Inbody measurement system is not a gold standard tool to assess body composition. However, it was reported that visceral fat measurements by InBody 720, which is an identical method to that used in our study, are highly correlated with those measured by abdominal CT scan, a gold standard method to measure visceral abdominal fat (*r* = 0.759) [20]. Lastly, it was reported that commercial ELISA kits could be not an accurate measure of serum irisin levels due to non-specific cross-reactivity [21]. For instance, median irisin levels in our study were somewhat higher (1.71 μg/mL) than those measured in other studies (0.76–1.03 μg/mL) using identical ELISA kits [10, 22]. Therefore, our results should be confirmed with other more valid methods to measure serum irisin levels.

## Conclusions

Circulating irisin is correlated to favorable metabolic profile, including reduced obesity, lower blood pressure and lower glucose levels, healthy lipid parameters, and increased insulin sensitivity. Irisin was also inversely associated with the diagnosis of pre-diabetes or T2D, independent of other well known risk factors for T2D. However, the function of irisin is more important than is the circulating irisin level, with regard to the metabolic effects and body composition of skeletal muscle and visceral fat play key roles in its functionality.

## Additional file

10.1186/s12933-015-0319-8 Correlation between serum irisin and metabolic parameters.
