# Supplementary material for: The ratio of skeletal muscle mass to visceral fat area is a main determinant linking circulating irisin to metabolic phenotype
Source: Cardiovasc Diabetol. 2016 Jan 20;15:9. doi: 10.1186/s12933-015-0319-8 (PMC4719696; doi:10.1186/s12933-015-0319-8)
Supplement: Supplementary file 1 — 10.1186/s12933-015-0319-8 Correlation between serum irisin and metabolic parameters. [file 12933_2015_319_MOESM1_ESM.docx]

**Table S1** Correlation with serum irisin levels according to skeletal muscle mass to waist circumference tertile

|  | **Total**  **(n=424)** | **Lower tertile (n=142)** | **Middle tertile (n=141)** | **Upper tertile (n=141)** |
| --- | --- | --- | --- | --- |
| Age | -0.17**^**^** | -0.17^*^ | -0.21**^*^** | -0.11 |
| Body mass index | -0.10**^*^** | -0.07 | -0.06 | -0.16 |
| Systolic blood pressure | -0.11**^*^** | -0.10 | -0.04 | -0.19^*^ |
| Diastolic blood pressure | -0.14**^**^** | -0.06 | -0.11 | -0.25**^**^** |
| OGTT (0 min) | -0.25**^†^** | -0.08 | -0.30**^**^** | -0.31^**^ |
| OGTT (120 min) | -0.00 | 0.06 | -0.002 | -0.06 |
| A1c | -0.05 | 0.06 | -0.12 | -0.09 |
| HOMA-IR | -0.18^†^ | -0.10 | -0.28**^**^** | -0.19^*^ |
| HOMA-B% | 0.01 | -0.05 | 0.05 | 0.004 |
| Total cholesterol | -0.16**^**^** | -0.05 | -0.24^**^ | -0.18**^*^** |
| Triglycerides | -0.21**^†^** | -0.01 | -0.25**^**^** | -0.37^**^ |
| HDL cholesterol | 0.13**^**^** | 0.09 | 0.08 | 0.21**^*^** |
| LDL cholesterol | -0.10 | -0.03 | -0.12 | -0.15 |
| High-sensitivity CRP | 0.12**^*^** | 0.08 | 0.15 | 0.12 |

^*^*P*<0.05, ^**^*P*<0.01, ^†^*P*<0.001

**Table S2** Correlation analysis with serum irisin levels according to the presence of metabolic syndrome

|  | Metabolic syndrome (-) | Metabolic syndrome (+) |
| --- | --- | --- |
| Age | -0.29^†^ | 0.01 |
| Body mass index | -0.14^*^ | 0.11 |
| Waist circumference | -0.14^*^ | -0.01 |
| Systolic blood pressure | -0.06 | -0.06 |
| Diastolic blood pressure | -0.12 | -0.11 |
| OGTT (0 min) | -0.38^†^ | 0.08 |
| OGTT (120 min) | -0.07 | 0.10 |
| A1c | -0.19^**^ | 0.20^*^ |
| HOMA-IR | -0.32^†^ | 0.16^*^ |
| HOMA-B% | -0.05 | 0.15 |
| Total cholesterol | -0.22^†^ | -0.01 |
| Triglycerides | -0.22^†^ | -0.18^*^ |
| HDL cholesterol | 0.08 | 0.10 |
| LDL cholesterol | -0.16^*^ | 0.01 |

^*^*P*<0.05, ^**^*P*<0.01, ^†^*P*<0.001
